# CRISP: A cost-efficient, high-throughput pipeline for sex-identification in *Cannabis sativa*

**DOI:** 10.17912/micropub.biology.001891

**Published:** 2025-12-17

**Authors:** Hyuk Sung Yoon, Mark Zander

**Affiliations:** 1 Waksman Institute of Microbiology, Rutgers, The State University of New Jersey, New Brunswick, New Jersey, United States

## Abstract

Hemp ( *Cannabis sativa* ) is a dioecious species whose glandular trichomes, found predominantly on female flowers, produce a variety of phytocannabinoids. Determining plant sex during the vegetative stage is not feasible, as only flowering plants allow accurate distinction. To efficiently utilize cultivation space and prevent unwanted pollination, it is therefore essential to reliably identify male hemp plants at an early developmental stage. Although numerous methods for sex identification in hemp have been reported, most rely on multi-step DNA extraction workflows and require costly equipment and consumables. Here, we present CRISP (Cannabis Rapid Identification of Sex by PCR), a simple, rapid, and cost-effective high-throughput approach. CRISP combines a simplified genomic DNA extraction via high-temperature chemical lysis with PCR detection of the male hemp marker SCAR323 in a 96-well format. We identified male seedlings from two cultivars with 100% accuracy, demonstrating the suitability of CRISP for large-scale sex identification in a highly cost-efficient manner.

**Figure 1. Development of the CRISP pipeline for sex identification:**
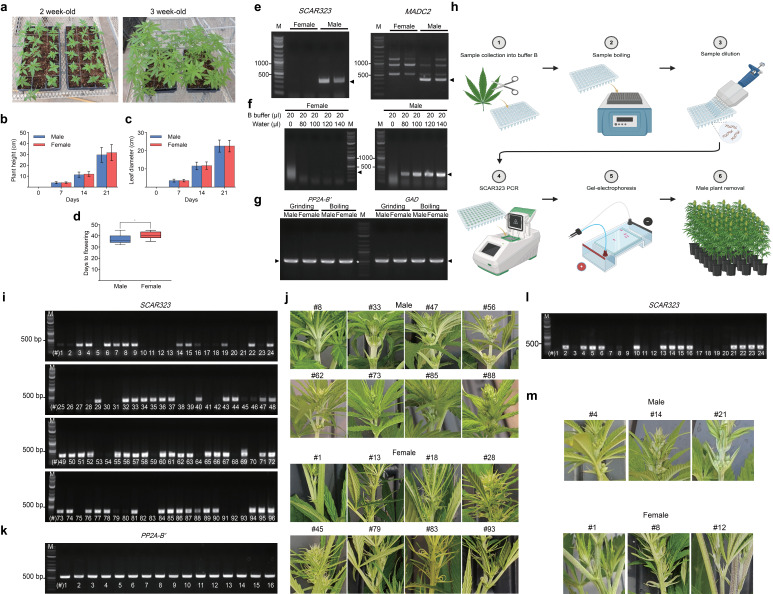
(a) Growth phenotypes of two- and three-week-old plants are shown. (b), (c) Quantification of plant height (b) and leaf length (c) of four-week-old male and female plants is shown. Data are presented as means ± standard deviation (SD) (n = 16 for male plants and 20 for female plants). (d) Quantification of flowering time is indicated (* Indicates statistical significance with *p* < 0.05 compared to male plants, as determined by Student’s *t* -test). (e) Agarose gel pictures show successful amplification of two male-specific DNA markers using primers against *SCAR323* (323 bp amplicon, left panel) and *MADC2* (390 bp amplicon, right panel). Genomic DNA was extracted from fan leaves of two-month-old female and male Otto II BOAX plants using the Edwards method. (f) Agarose gel images show the absence of SCAR323 amplicons in DNA from female plants (left panel) and their presence in DNA from male plants (right panel). In addition, various dilutions with ultrapure water at the indicated volumes were tested. (g) Agarose gel picture shows PCR results from a comparison between DNA extracted with the Edwards method and through high-temperature chemical lysis using the housekeeping genes *PP2A-B′* (555 bp amplicon) and *GAD* (578 bp amplicon). The DNA ladder is labeled as ‘M’ in all panels, and the white asterisk indicates the 500 bp band on the ladder. (h) Various steps of the CRISP pipeline are outlined. Step 1: Collect 2–3 mg of fan leaf tissue from two-week-old hemp seedlings and immerse the samples in 20 μl of B buffer in a 96-well plate. Step 2: Heat the plate at 100 °C for 10 minutes on a heat block. Step 3: Dilute each sample with 100 μl of ultrapure water using a multi-channel pipette. Step 4: Add 1 μl of the extracted solution to 11 μl of Taq DNA polymerase PCR master mix containing SCAR323 primers. Step 5: Load the PCR products onto a 1% agarose gel to visualize the SCAR323 amplicon. Step 6: Discard male seedlings, and transfer female plants to larger pots for continued growth. (i) Agarose gel picture shows the result of a PCR analysis using SCAR323 primers (323 bp amplicon) from 96 two-week-old Otto II BOAX seedlings as part of the CRISP pipeline. The DNA ladder is indicated as 'M' in the figure. (j) Photographs were taken from flowers to confirm the presence or absence of male flowers. PCR-confirmed flower phenotypes of eight male and eight female plants are shown, with plant line numbers corresponding to those presented in panel (i). (k) Agarose gel picture shows PCR results from 16 DNA samples using the housekeeping gene *PP2A-B′* (555 bp amplicon). The DNA ladder is indicated as 'M' in the figure. Plant line numbers correspond to those presented in panel (i). (l) Agarose gel image showing the results of the CRISP pipeline used to detect male seedlings among 24 two-week-old Cherry #4 seedlings, using SCAR323 primers (323 bp amplicon). The DNA ladder is indicated as 'M' in the figure. (m) Photographs were taken from flowers to confirm the presence or absence of male flowers. PCR-confirmed flower phenotypes of three male and three female plants are shown, with plant line numbers corresponding to those presented in panel (k). Parts of panel (h) were created with BioRender (https://BioRender.com/g4jl8ch).

## Description

Hemp ( *Cannabis sativa* ) is an economically important crop primarily for the production of valuable phytocannabinoids, as well as for fiber and seeds (Fordjour et al., 2023; Viskovic et al., 2023; Górski et al., 2025). It is a dioecious plant species with nine pairs of autosomes and one pair of sex chromosomes (2n = 20) (Prentout et al., 2020). Sexual characteristics in hemp are controlled by an X-to-autosome balance mechanism, with males carrying heteromorphic sex chromosomes (XY) and females carrying XX (Razumova et al., 2016). Remarkably, hemp was among the first domesticated plants in the early Neolithic period in East Asia (Ren et al., 2021). Its controversial reputation stems from the presence of more than 100 phytocannabinoids, with THC (tetrahydrocannabinol) and CBD (cannabidiol) being the most prominent (Gulck and Moller, 2020). Importantly, phytocannabinoids are produced in specialized structures known as glandular trichomes, which are concentrated mainly on the flower buds of female plants (Tanney et al., 2021; Hancock et al., 2024). As a result, only female plants are cultivated, highlighting the importance of an efficient sex identification system to detect and remove male hemp seedlings at an early developmental stage.

Over the past three decades, multiple sex determination markers have been identified in *Cannabis sativa* using various molecular approaches. RAPD (Random Amplification of Polymorphic DNA) analyses led to the discovery of MADC (Male-Associated DNA from *C. sativa* ) markers, MADC1 - MADC6 (Sakamoto et al., 1995; Mandolino et al., 1999; Törjék et al., 2002; Sakamoto et al., 2005). An AFLP (Amplified Fragment Length Polymorphism) study further identified twenty primer combinations that can amplify male-specific DNA fragments (Flachowsky et al., 2001; Peil et al., 2003). RAPD markers can be converted into SCAR (Sequence Characterized Amplified Region) markers by excising and sequencing RAPD fragments, then designing locus-specific primers to create reproducible PCR (polymerase chain reaction) assays. This strategy was applied to MADC2, enabling early identification of male hemp plants via PCR (Techen et al., 2010; Faux et al., 2014; Mendel et al., 2016; Razumova *et al.* , 2016; Nalesnik and Erickson, 2020). Similarly, MADC5 and MADC6 were converted into SCAR323 and SCAR119 markers, which also reliably identified male plants using classical PCR (Törjék *et al.* , 2002; Mendel *et al.* , 2016; Razumova *et al.* , 2016; Nalesnik and Erickson, 2020; Borin et al., 2021). The robustness of MADC2 was further demonstrated in a large-scale study genotyping 8,200 hemp seedlings with real-time qPCR, which also tested alternative methods using MADC2 such as LAMP (Loop-mediated Isothermal Amplification), RPA (Recombinase Polymerase Amplification), and HRM (High-Resolution Melting) analysis (Torres et al., 2022). HRM analysis has also been employed to distinguish male and female hemp plants using two sex-specific SNPs in the *SEUSS* corepressor gene (Gilchrist et al., 2023).

Additionally, MADC6 was used for the development of CSP-1 (Cannabis Sex Primer-1), a high-throughput PACE (PCR Allele Competitive Extension) assay for sex identification (Toth et al., 2020). Recently, a multiplex PCR approach combining two Y-specific coding-region markers and one autosomal control marker was introduced, based on the prior identification of 347 expressed Y-linked genes (Prentout *et al.* , 2020; Prentout et al., 2025). Another novel marker, CsPDS5 (PRECOCIOUS DISSOCIATION OF SISTERS 5), has been incorporated into a CsPDS5-CAPS (Cleaved Amplified Polymorphic Sequence) TaqMan assay for accurate detection of male hemp plants (Riera-Begue et al., 2025).

While these various sex determination approaches are highly accurate, the experimental pipeline can be lengthy and costly, requiring specialized equipment beyond a simple thermocycler, as well as fluorescent probes and expensive DNA polymerase master mixes (Toth *et al.* , 2020; Torres *et al.* , 2022; Riera-Begue *et al.* , 2025). The protocol’s complexity stems from genomic DNA extraction and the selection of subsequent sex identification assays. Extraction of genomic DNA is usually achieved with DNA extraction kits or more commonly with variations of the CTAB (Cetyltrimethylammonium bromide) method (Doyle, 1987; Toth *et al.* , 2020; Riera-Begue *et al.* , 2025). The CTAB method is cost-efficient but relatively complex, involving multiple steps such as DNA separation with chloroform-isoamyl alcohol and DNA precipitation using ethanol or isopropanol, which can hinder its high-throughput capacity (Doyle, 1987). Sex identification from extracted genomic DNA can subsequently be performed using a classical PCR approach that visualizes sex-specific amplicons on agarose gels (Törjék *et al.* , 2002; Mendel *et al.* , 2016; Razumova *et al.* , 2016; Nalesnik and Erickson, 2020; Borin *et al.* , 2021), or by applying fluorescent probe-based methods such as real-time qPCR, the CSP-1 PACE assay, or the CsPDS5-CAPS TaqMan assay (Toth *et al.* , 2020; Torres *et al.* , 2022; Riera-Begue *et al.* , 2025).

Here, we report the development of CRISP (Cannabis Rapid Identification of Sex by PCR), a cost-effective high-throughput sex identification workflow for hemp seedlings. By streamlining genomic DNA extraction through high-temperature chemical lysis, sample processing is reduced to just two steps before sex identification via PCR detection of the SCAR323 marker. CRISP is implemented in a 96-well format, enabling rapid, large-scale identification of male hemp seedlings.

**Simplifying extraction of genomic hemp DNA through high-temperature chemical lysis**

Most sex identification assays in hemp utilize the CTAB method which relies on the physical disruption of frozen plant tissue via grinding and the subsequent multi-step purification of DNA. To simplify the DNA extraction procedure, we tested whether genomic DNA from hemp can be successfully extracted through a rapid two-step high-temperature chemical lysis protocol. This protocol solely relies on the boiling of samples in extraction buffer and subsequent dilution with water (Tsugama *et al.* , 2011). Primers against two well-characterized male-specific markers, SCAR323 and MADC2, were used to test the suitability of extracted DNA for sex identification through PCR (Mandolino *et al.* , 1999; Törjék *et al.* , 2002; Razumova *et al.* , 2016). For our analyses, we used two-week-old Otto II BOAX plants, which exhibited no discernible sex-specific differences in plant morphology, height or leaf length (Figure 1a-d). Male flowers emerged after 32-45 days, while female flowers appeared slightly later, between 35 and 45 days, allowing reliable visual sex identification (Figure 1d).

Genomic DNA from mature male and female Otto II BOAX leaf tissue was extracted with the Edwards method and via high-temperature chemical lysis. *SCAR323* (323 bp amplicon) and *MADC2* (390 bp amplicon) amplicons were successfully detected in DNA of male samples derived from both extraction methods and were, importantly, absent in female samples (Figure 1e). Since MADC2 PCR produced three extra amplicons, whereas SCAR323 produced none (Figure 1e), we chose SCAR323 for further analyses. To test whether sample dilution with water is needed, we also compared undiluted with diluted samples and found that only the diluted male samples show the *SCAR323* amplicon (Figure 1f). Diluted samples consistently produced a clear male-specific amplicon, whereas undiluted DNA from both male and female plants displayed smearing (Figure 1f). In addition, to validate the genomic DNA prepared by both extraction methods, we also performed PCR to amplify two *Cannabis* reference genes, *PP2A-B’* (555 bp amplicon) and *GAD* (578 bp amplicon). All samples showed the expected amplicon size (Figure 1g), indicating that genomic DNA extraction through chemical lysis is suitable for PCR-based sex identification in hemp.

**Identifying male hemp plants at scale using CRISP**

To conduct sex identification of hemp seedlings in a high-throughput manner, we developed the CRISP pipeline, which adapted the high-temperature chemical lysis protocol for use in a 96-well format (Figure 1h). CRISP successfully detected 58 *SCAR323* amplicons among 96 two-week-old Otto II BOAX seedlings (Figure 1i). To evaluate its accuracy, all 96 plants were grown to full flowering, and the emergence of male flowers was recorded (Extended Data Table 1). Every seedling exhibiting a male-specific *SCAR323* signal also produced male flowers (Figure 1g), while the remaining plants developed female flowers, demonstrating the high reliability of the CRISP pipeline. Additionally, we confirmed DNA quality from the CRISP protocol using PP2A-B′ primers (Figure 1k). To further assess the robustness of CRISP, we analyzed 24 seedlings from another hemp cultivar, Cherry #4. This analysis identified 13 putative male plants based on *SCAR323* amplicons, which were later validated by the presence of male flowers at maturity (Figure 1l,m, and Extended Data Table 2).

Although feminized seeds are commercially available, they are typically more expensive than non-feminized seeds and must be purchased each season, as they are produced through a complex feminization process of female flowers. Consequently, large-scale early-stage sex identification represents a promising alternative. However, the adoption of biotechnological methods in agriculture and breeding is often limited by the complexity and cost of existing pipelines. To address this, we designed the CRISP pipeline to be as simple and cost-effective as possible. Unlike previously reported approaches, CRISP functions effectively with basic Taq polymerase and does not require fluorescence-based probes or a real-time PCR cycler. The estimated cost of a 96-sample CRISP run is approximately $6, whereas most commercially available sex-identification kits range between $8 and $15 per sample. The entire CRISP pipeline takes approximately 4 hours, including sample collection (1 hour), genomic DNA extraction (0.5 hours), PCR (1.5 hours), and gel electrophoresis (0.5 hours). This duration is comparable to, or slightly shorter than, other methods; however, a key advantage of CRISP is that it can be performed with up to 96 samples in parallel. Thus, CRISP represents a rapid, reliable, and cheap approach for sex-identification in hemp.

Another promising area of application is chemotype determination in hemp. The chemotype generally refers to the ratios of key phytocannabinoids such as THC, CBD, and CBG (cannabigerol). Classification distinguishes between several types: THC-dominant (Type I), balanced CBD/THC (Type II), CBD-dominant or “true hemp” (Type III), CBG-dominant (Type IV), and the null type (Type V) (Aizpurua-Olaizola et al., 2016). Among these, THC content is particularly critical, as it defines the legal status of a given hemp cultivar. Historically, hemp cultivation and use were prohibited - and in some regions remain restricted - under regulations linked to THC levels. Even in countries where chemotypes I and II are prohibited (drug-type) and chemotypes III, IV, and V (industrial-type) are permitted, routine testing of THC levels is often required to ensure compliance with regulatory standards. The enzymes THCAS (tetrahydrocannabinolic acid synthase) and CBDAS (cannabidiolic acid synthase) determine whether a hemp cultivar predominantly produces THC, CBD, both, or neither (Melzer et al., 2022). PCR-based detection of the *THCAS* gene offers a promising approach to differentiate between drug-type and industrial hemp. However, THCAS and CBDAS genes exhibit high sequence similarity and are embedded within a highly complex genomic locus, characterized by long terminal repeat retrotransposons as well as variable numbers of THCAS/CBDAS genes and pseudogenes (Weiblen et al., 2015; Laverty et al., 2019; Grassa et al., 2021; Lynch et al., 2025). Recently, novel sets of primers were identified that can reliably distinguish between THCAS and CBDAS (Forlani and Petrollino, 2021; Boonjing et al., 2025), and these could be integrated into our CRISP pipeline. In summary, CRISP is a versatile tool with broad applicability to analyses that depend on distinguishing PCR amplicon size or detecting amplicon presence versus absence.

## Methods

**Plant materials and growth conditions**

Two *Cannabis sativa* cultivars, Otto II BOAX and Cherry #4, were obtained from Colorado Breeders Depot (Cañon City, Colorado, USA). Seeds were sown in 18-pot trays (8 cm × 8 cm per pot) and grown in a greenhouse maintained at 25 °C under a 16-hour light/8-hour dark photoperiod. Plants were watered regularly with tap water and fertilized weekly with CANNA Terra Vega (Arcadia, California, USA). To compare growth between male and female plants, plant height and total leaf length were recorded weekly. Flowering time was assessed by observing plants daily at 10:00 AM for the emergence of male flowers and female flowers. The growth, handling, and processing of hemp materials were performed under Rutgers University’s licenses for hemp cultivation (34_00011), processing (34_00046), and handling (34_00047), issued by the New Jersey Department of Agriculture.

**Genomic DNA extraction**

For the initial validation of MADC2 and SCAR323 primers we used genomic DNA that was extracted with Edwards protocol (Edwards et al., 1991). Primer sequences are as follows: SCAR323_Fw: GAGCGGACATCATTGCCT, SCAR323_Rv: ATCACCCCACCGTTTAGG, MADC2_Fw: GTGACGTAGGTAGAGTTGAA, MADC2_Rv: GTGACGTAGGCTATGAGAG, PP2A-B *′* _Fw: GCTCGAAATCCACCTCTGCT, PP2A-B *′* _Rv: TGGCTAGCTTGGCATCAGTC, GAD_Fw: GGGCTCCAACCAATTTCCCA, GAD_Rv: ATCAGACCCTCGACAATGCC. In this protocol, 2-3 mg of liquid nitrogen-frozen fan leaf tissue was ground in 200 µl of extraction solution containing 200 mM Tris-HCl (pH 7.5), 250 mM NaCl, 25 mM EDTA, and 0.5% (w/v) SDS in 1.5 ml microcentrifuge tubes. The homogenates were centrifuged at 14,000 × *g* for 2 min, and the supernatants were transferred to fresh 1.5 ml tubes. A total of 150 µl of isopropanol was added, followed by centrifugation at 14,000 × *g* for 5 min. After discarding the supernatants, 400 µl of 70% ethanol was added, and the samples were centrifuged again at 14,000 × *g* for 1 min. The resulting pellets were dried and resuspended in 30 µl of sterilized ultrapure water. Finally, 0.8 µl of the supernatant was used for PCR reactions.

**CRISP pipeline**

High-temperature chemical lysis was performed as described previously with some modifications (Tsugama et al., 2011). Plant samples (2-3 mg) were collected into a 96-well plate and submerged into 20 µl of B (boiling) buffer containing 50 mM Tris-HCl, pH 8.0, 50 mM EDTA, and 0.2% (w/v) SDS in a 96-well plate. Most steps were performed with multichannel pipettes. The plate was covered with a heat-resistant sealing film and incubated at 100°C for 10 min on a heat block. Samples were precipitated by brief centrifugation at 1,000 g for 1 min and then diluted with sterilized 100 µl ultrapure water. 1 µl of the supernatant was used for the subsequent PCR analysis using primers that amplify the male-specific DNA markers MADC2 and SCAR323. These primers were previously described (Mandolino *et al.* , 1999; Törjék *et al.* , 2002; Razumova *et al.* , 2016). Amplification of two *Cannabis sativa* housekeeping genes *PP2A-B’* ( *PROTEIN PHOSPHATASE 2A B′ subunit)* (Accession number: XM_030625838) and *GAD* ( *GLYCERALDEHYDE-3-PHOSPHATE DEHYDROGENASE* ) (Accession number: XM_030636658) were used for DNA quality control (Deguchi et al., 2021).

For all PCRs conducted as part of the CRISP pipeline, each reaction contained 6 µl of 2× PCR PreMix (Green Dye) (MB067-EQ2G-L, Syd Labs, USA), 1 µl of template DNA, 1 µl of each 10 µM primer, and 3 µl of ultrapure water, for a total volume of 12 µl per sample. Amplifications were performed on a Bio-Rad C1000 Thermal Cycler with the following program: 94 °C for 5 min; 35 cycles of 94 °C for 30 sec, 62 °C for 30 sec, and 72 °C for 45 sec; followed by a final extension at 72 °C for 7 min. PCR products were analyzed by gel electrophoresis on a 1% agarose gel.

## Data Availability

Description: Extended Data Table. Resource Type: Dataset. DOI: https://doi.org/10.22002/a7btq-nhm40
